# Comparison of two different methods for lymphadenectomy along the left recurrent laryngeal nerve by minimally invasive esophagectomy in patients with esophageal squamous cell carcinoma: a prospective randomized trial

**DOI:** 10.1097/JS9.0000000000000788

**Published:** 2023-09-21

**Authors:** Ying-Jian Wang, Xian-Dong He, Yi-Qiu He, Tao Bao, Xian-Feng Xie, Kun-Kun Li, Wei Guo

**Affiliations:** a Department of Thoracic Surgery, Army Medical Center of PLA (Daping Hospital), Daping; b Department of Pediatrics, Shapingba District Maternity and Infant Health Hospital, Shapingba, Choingqing, People’s Republic of China

**Keywords:** complications, esophageal cancer, left recurrent laryngeal nerve, lymphadenectomy, minimally invasive esophagectomy, quality of life, survival

## Abstract

**Background:**

Lymph nodes along the left recurrent laryngeal nerve (LRLN) is thought to be highly involved in esophageal cancer. Given the unique anatomical positioning of the nerve, performing lymphadenectomy in this region requires advanced techniques within limited working space. Meanwhile, high incidence of morbidity and mortality is associated with lymphadenectomy. Although several methods have been applied to reduce the technical requirement and the incidence of postoperative complication, the optimal method remains controversial.

**Methods:**

This study was a single-center, prospective, randomized trial to investigate the utility of lymphadenectomy along the LRLN during the minimally invasive esophagectomy in esophageal squamous cell carcinoma patients by comparing the surgical outcome, postoperative complication, survival rate, and quality of life (QoL) between the retraction method (RM) and the suspension method (SM) in patients with esophageal cancer from June 2018 to November 2020. QoL was assessed according to questionnaire: EQ-5D-5L.

**Results:**

Of 94 patients were enrolled and randomized allocated to RM and SM group equally. Characteristics did not differ between groups. The duration of lymph node dissection along LRLN was significant longer in SM group (*P*<0.001). No difference was observed about postoperative complications. One of in-hospital death was occurred in each group (*P*>0.999). Patients in neither of groups exhibiting difference about 3-year disease-free survival rate (*P*=0.180) and overall survival rate (*P*=0.430). No difference was observed in postoperative QoL between groups at different time points (all, *P*>0.05).

**Conclusion:**

Both methods of lymph node dissection along the LRLN during minimally invasive esophagectomy in esophageal squamous cell carcinoma patients are technically feasible and safe. The RM appears more favorable in terms of reducing surgical duration compared to the SM.

## Introduction

Highlights

- The first published prospective randomized controlled trial demonstrated that both retraction method and suspension method in lymphadenectomy along the left recurrent laryngeal nerve (LRLN) showed favorable outcomes and a similar safety profile.
- Survival rate of different methods of lymphadenectomy along the LRLN was evaluated.
- Quality of life assessment was carried out between different lymphadenectomy methods along the LRLN for the first time.

Lymph node metastasis is the most important prognostic factor for esophageal cancer^1^. High quality of lymphadenectomy is critical for local disease control and possibly enhances survival^2^. According to the pattern of lymph node metastasis in esophageal cancer, upper mediastinum is thought to be highly involved^3^. During esophagectomy conducted with curative intent, a thorough and efficacious dissection within this region assumes a pivotal role. Nevertheless, the anatomical positioning of the left recurrent laryngeal nerve (LRLN) imposes limitations on available workspace for lymphadenectomy, particularly within the framework of minimally invasive procedures necessitating advanced surgical techniques. This challenge is compounded by the elevated morbidity rates linked to lymphadenectomy in this region, potentially exerting an adverse impact on both prognosis and the quality of life (QoL)^4^. Therefore, many different methods, such as ʻbascule methodʼ^5^, ʻstripping methodʼ^6^, and ʻmagnetic anchoring methodʼ^7^, were reported with the intention of reducing the risk of iatrogenic LRLN injury. Nonetheless, each of method has its pros and cons, and a consistent surgical strategy has not yet been established. Therefore, we designed a prospective randomized clinical trial in comparing two different methods, the retraction method (RM) and suspension method (SM), with the aim of assisting surgeons in making more informed treatment-related decisions.

## Materials and methods

### Study design

This study was a single-center, prospective, randomized trial (ChiCTR1800016963, http://www.chictr.org.cn/edit.aspx?pid=28828&htm=4) to investigate the difference among surgical outcome, postoperative complication, survival rate, and QoL between RM and SM for lymphadenectomy along LRLN in patients with esophageal squamous cell carcinoma (ESCC). Patients were recruited from June 2018 to November 2020 in Army Medical University of PLA (Daping hospital). This study was conducted in conformance with Good Clinical Practice guidelines and the Declaration of Helsinki and approved by the ethics committee of the Army Medical Center of PLA (Daping Hospital) (Number: 201816). The research has been reported in line with the Consolidated Standard of Reporting Trials (CONSORT) Guidelines^8^. Written informed consent was obtained from the patient for the publication of this case report and accompanying images. A copy of the written consent is available for review by the Editor-in-Chief of this journal on request. The data were recorded prospectively in an electronic database.

### Patient eligibility

Patients aged 18–75 years with histologically confirmed ESCC without distant metastasis and who did not participate in other clinical trials were eligible for this trial. Patients were ineligible if they had any significant medical condition which was thought unlikely to tolerate the surgery, such as active tuberculosis, liver dysfunction, renal function disorder, and another malignant tumor. Patients who had a history of gastrointestinal perforation, fistula, or thrombus within 6 months before surgery, had a mental disorder or history of psychotropic drug abuse, or had a major operation within 4 weeks before surgery were also ineligible. Patients with long-term unhealed wounds or fractures, significant malnutrition, as well as pregnant or lactating patients at screening were also excluded from this trial.

### Procedures

Eligible patients were randomly (allocation ratio 1:1) assigned to undergo lymphadenectomy along the LRLN by RM or SM via a computer-generated coding system. The block sizes will be chosen by the statistician so that each block contains the patients in equal proportion. This procedure helps to ensure both randomness and investigator blinding (the block sizes are known only to the statistician). If patients received neoadjuvant chemotherapy, MIE was scheduled 4–6 weeks after the completion of therapy. Preoperative restaging is comprised of a physical examination, standard laboratory tests, esophagogastroduodenoscopy with endoscopic ultrasound, pulmonary function tests, and neck-thorax-abdomen computed tomography. All the surgeries were performed by a single surgeon (Dr Wei Guo), who had performed more than 1000 MIE prior to this study.

Following the general anesthesia with the placement of a single-lumen endotracheal tube, the patient was placed in the semi-prone position. Four 5- or 12 mm trocars were placed in the fourth and sixth intercostal space (ICS) in the midaxillary line, the eighth ICS in the posterior axillary line and the sixth ICS in the scapular angle line. Carbon dioxide (CO_2_) insufflation was used at a pressure of 6–8 mmHg to depress the diaphragm caudally and lung anteriorly, maximizing exposure of the intrathoracic esophagus. In the beginning, common to both methods, the mediastinal pleura overlying the anterior and posterior aspect of middle and lower esophagus was opened by the hook electrocautery, mobilizing the esophagus from the crura to the level of an azygous vein, which was skeletonized, clipped, and transected by a harmonic scalpel. Then, opening the upper mediastinal pleura overlying the anterior aspect of the esophagus along the right vagus nerve until extending to the intersection of the vagus nerve and right subclavian artery. The right recurrent laryngeal nerve was identified and exposed carefully by using the separating forceps and lymph nodes with adipose tissues along the right recurrent laryngeal nerve were dissected.

**Figure 1:**
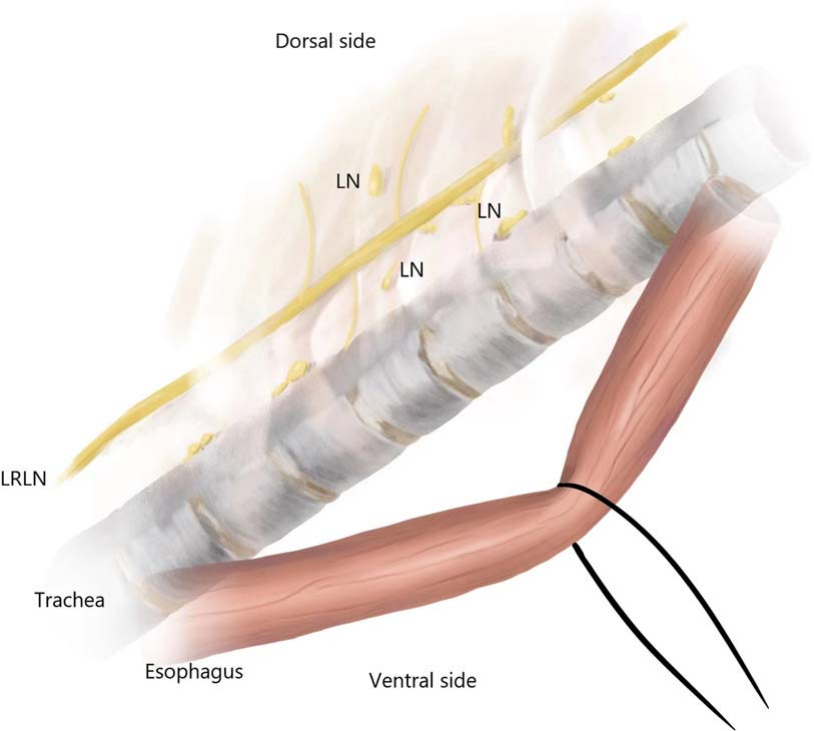
Schematic drawing of lymphadenectomy along the LRLN by retraction method. LRLN, left recurrent laryngeal nerve; LN, lymph node.

After then, in the RM group, the esophagus was completely mobilized and freed from the trachea and retracted to the anterior aspect of the chest by a loop to maximize the exposure of the dorsal side of the upper mediastinum. Within a clockwise rotation of the trachea ventrally using blunt forceps by an assistant, the tracheoesophageal groove can be visualized. To identify the LRLN, the lymph nodes (station 4L) beneath the aortic arch were first scavenged. The operator used a grasp forceps by the left hand to lift the lymph nodes and surrounding tissues, and another forceps was used by the right hand to separate the tissues bluntly to identify the LRLN. Once the LRLN was visualized, a meticulous dissection was performed, encompassing the separation of lymph nodes and adjacent tissue from the upper margin of the left main bronchus to the base of the aortic arch. Subsequently, the process extended further, lifting lymph nodes and surrounding tissue from the upper margin of the aortic arch to the base of the left thyroid gland, executed via grasp forceps. An additional forceps was then utilized to cautiously dissect the lymph node and adjacent tissue from the LRLN using blunt maneuvers. Identification of the tracheoesophageal arteries prompted the utilization of an ultrasound scalpel for their precise transection. Simultaneously, branches of the LRLN were meticulously severed using scissors to avert nerve injury. Ultimately, lymph nodes situated along the LRLN, alongside associated tissues, were meticulously excised and released from the esophagus (Fig. 1).

**Figure 2:**
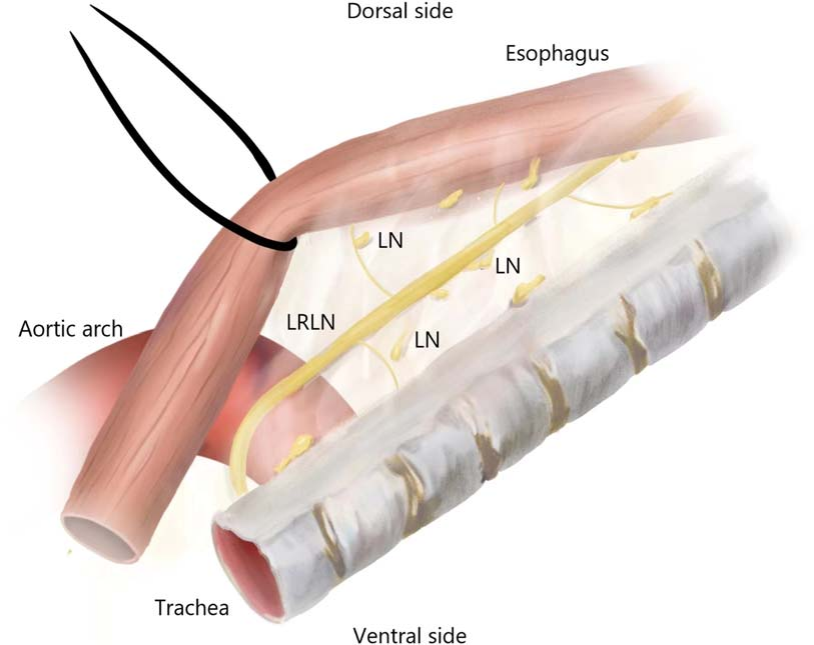
Schematic drawing of lymphadenectomy along the LRLN by suspension method. LRLN, left recurrent laryngeal nerve; LN, lymph node.

In SM group, the upper mediastinal pleura overlying on the posterior aspect of the esophagus is opened first to delineate the dorsal border of the dissected plane. Next, the lateral pedicle including lymph nodes, adipose tissues and LRLN was dissected sharply just along the trachea and the left bronchus with the esophagus to delineate the ventral border of the dissected plane. After then, the proximal esophagus is encircled with a loop, which is held on the chest wall, to maximize the exposure of the tracheoesophageal groove. Now a membrane-like (lateral pedicle) structure is extended between the esophagus and trachea, which includes lymph nodes, adipose tissue, LRLN, and tracheoesophageal arteries, and can be recognized easily. Consequently, an operator can use both hands to blunt and sharply separate lymph nodes from LRLN. Similarly, the dissection border was from the upper edge of the left main bronchus to the lower edge of the inferior thyroid arteries. Different from RM, after dissection, lymph node, and adipose tissue were attached to the esophagus (Fig. 2).

### Endpoints

Evaluation of the techniques includes surgical duration for lymphadenectomy along the LRLN and for total and entire thoracic procedure; number of retrieved lymph nodes along the LRLN; estimated blood loss during the lymphadenectomy along the LRLN; major intraoperative and postoperative morbidity related to the LRLN, such as aspiration and pneumonia. Disease-free survival (DFS; the time duration from the start of therapy to tumor recurrence or death from any cause), OS (the time duration from the start of therapy to death from any cause) and QoL were also assessed. An independent Data Monitoring Committee will be appointed to monitor the study and make decisions regarding possible early closure and publication.

### Follow-up

Patients were followed up every 3 months in the first 2 years, then every 6 months in the following 3 years, and then annually after 5 years. The duration of follow-up was calculated from the completion of treatment to the last contact or death. The final follow-up was on 21 January 2023.

## Theory/Calculation

### Statistical analysis

On the basis of our previous studies, sample size calculations were made assuming a surgical duration of lymphadenectomy along the LRLN of 25 min for patients assigned to the RM group and 30 min for those assigned to the SM group. With a two-sided type I error of 0.05 and a power of 80%, a randomization ratio of 1:1 between the experimental and control arms, the intended number of randomly assigned patients was 86 (43 per arm). Efficacy analysis was performed according to the per-protocol set, which was defined as all patients who completed MIE and/or neoadjuvant treatment without serious violation of the protocol. Safety was evaluated based on a safety analysis set, which defined all patients who completed the MIE, and at least one assessment of safety data. Categorical variables (expressed as numbers and percentages) between the two groups were compared using Fisher’s exact test or *χ*^2^ test, while continuous variables (expressed as mean with SD) were compared using the Mann–Whitney *U* test. The DFS and OS were calculated by the Kaplan–Meier method and compared using a stratified log-rank test. The MedCalc (MedCalc Software) software was used for statistical analysis. The significance level is set at *P*<0.05.

## Results

### Patient characteristics

**Figure 3:**
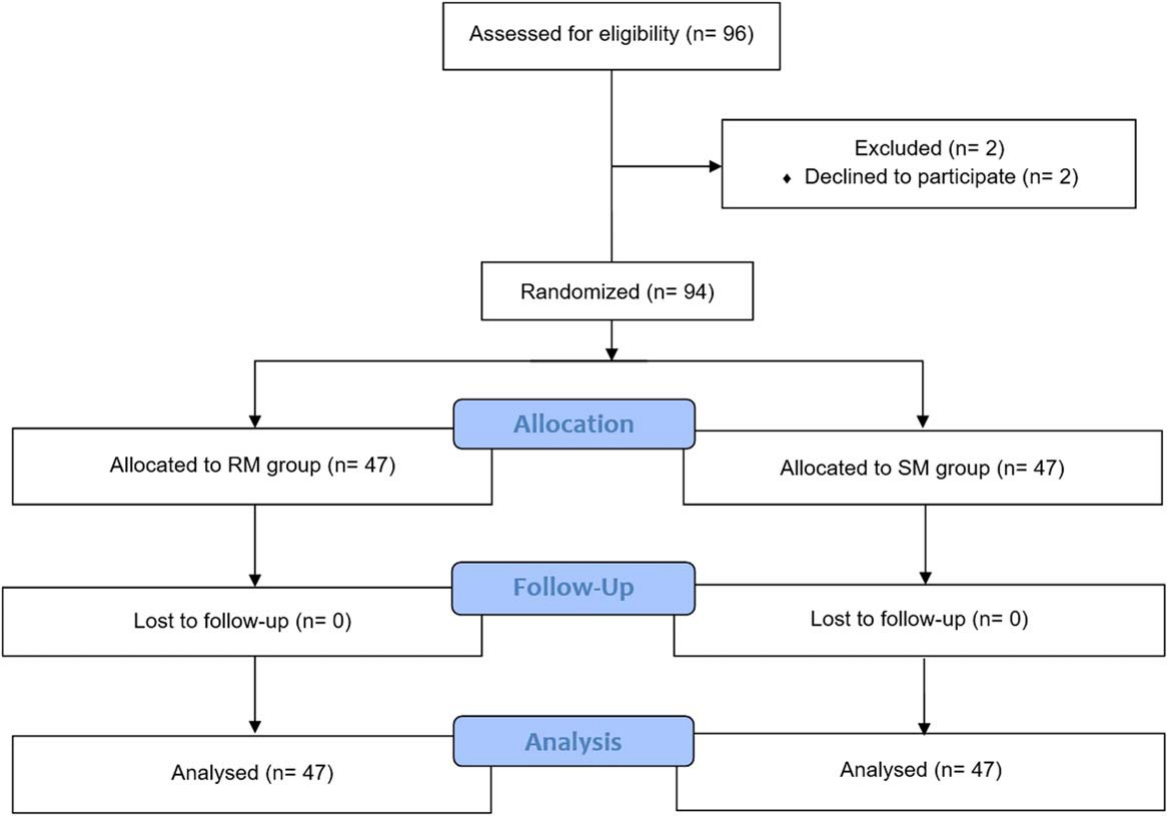
Study flowchart. RM, retraction method; SM, suspension method.

From June 2018 to November 2020, a total of 96 patients (Fig. 3) were assessed for eligibility, two of them refused to participate in the trial, finally, 94 patients were enrolled and randomized allocated to the RM group and SM group. Baseline characteristics (Table 1) including age, sex, BMI, smoking history, alcohol history, tumor location, and clinical T and N stage were generally well balanced between the two groups (all *P*>0.05).

**Table 1:** Baseline characteristics of two groups.

Characteristic	RM (*n*=47)	SM (*n*=47)	*P*
Age (years), median (range)	64.0 (53.0–82.0)	64.0 (47.0–76.0)	0.505
Sex (*n*, %)			>0.999
Male	37 (78.7)	37 (78.7)	
Female	10 (21.3)	10 (21.3)	
BMI (kg/m^2^, mean±SD)	21.6±3.0	22.7±2.8	0.487
Smoking history (*n*, %)	32 (68.1)	29 (61.7)	0.666
Alcohol abuse history (*n*, %)	24 (51.1)	26 (55.3)	0.836
Tumor location (*n*, %)			0.423
Upper	20 (42.6)	14 (29.8)	
Middle	18 (38.3)	23 (48.9)	
Lower	9 (19.1)	10 (21.3)	
T stage (*n*, %)			0.225
T1	19 (40.4)	12 (25.5)	
T2	8 (17.0)	7 (14.9)	
T3	20 (42.6)	28 (59.6)	
N stage (*n*, %)			0.878
N0	24 (51.1)	26 (55.3)	
N1	12 (25.5)	11 (23.4)	
N2	10 (21.3)	8 (17.0)	
N3	1 (2.1)	2 (4.3)	

n, number; RM, retraction group; SM, suspension group.

### Surgical outcome

MIE was performed for all 94 patients. Among them, all of the patients in both groups underwent R0 resection (*P*>0.05). The total operation duration (229.3±34.0 *vs* 231.6±37.7 min) was no significant difference between groups (*P*=0.961). When comparing the thoracic procedure, although the difference was not exhibited between groups, a trend of longer duration in the SM group was observed (89.3±21.0 *vs* 81.1±20.8 min, *P*=0.054), which contributed to the longer duration of LRNL dissection in SM group (25.0±4.1 *vs* 20.7±3.3 min, *P*<0.001). There was no significant difference between the two groups in terms of estimated blood loss during the LRLN dissection (*P*=0.842), conversion rate, retrieved lymph nodes along LRLN (total *P*=0.247, positive *P*=0.325) and intraoperative morbidity related to the LRLN (Table 2).

**Table 2:** Surgical outcomes of two groups.

Factors	RM (*n*=47)	SM (*n*=47)	*P*
Operation duration (min, mean±SD)			
Total duration	229.3±34.0	231.6±37.7	0.961
Thoracoscopic duration	81.1±20.8	89.3±21.0	0.054
LRLN dissection duration	20.7±3.3	25.0±4.1	<0.001
Blood loss during the LRLN dissection (ml, mean±SD)	11.5±5.5	11.4±5.3	0.842
Conversion to open surgery (*n*, %)	0	0	
Retrieved lymph nodes along LRLN (*n*, mean±SD)			
Total	5.1±1.5	5.3±1.3	0.247
Positive	0.2±0.4	0.2±0.7	0.325
Intraoperative morbidity related to the LRLN (*n*, %)	0	0	

LRLN, left recurrent laryngeal nerve; ml, milliliter; n, number; RM, retraction suspension; SM, suspension group.

### Postoperative complications

The most frequently observed complications related to the LRLN dissection were hoarseness (34 *vs* 19.1%, *P*=0.249), aspiration (10.6 *vs* 8.5%, *P*>0.999), pneumonia (29.8 vs 25.5%, *P*=0.818) and anastomotic leakage (12.8 *vs* 10.6%, *P*>0.999) in RM and SM group respectively. According to the Clavien–Dindo classification system, there was no significant difference between groups. The rates of reintubation were 4.3% (2/47) in the RM group, whereas 2.1% (1/47) in the SM group (*P*>0.999). One (2.1%) of in-hospital death was observed in each group (*P*>0.999) (Table 3).

**Table 3:** Postoperative complications of two groups.

Variables	RM (*n*=47)	SM (*n*=47)	*P*
RLN paralysis (*n*, %)	9 (19.1)	4 (8.5)	0.231
Aspiration (*n*, %)	5 (10.6)	2 (4.3)	0.435
Pneumonia (*n*, %)	14 (29.8)	12 (25.5)	0.818
Anastomotic leakage (*n*, %)	6 (12.8)	3 (6.4)	0.486
Clavien–Dindo grade (*n*, %)			
I	0	1 (2.1)	0.317
II	4 (8.5)	4 (8.5)	>0.999
IIIa	2 (4.3)	3 (6.4)	>0.999
IIIb	7 (14.9)	3 (6.4)	0.316
V	1 (2.1)	1 (2.1)	>0.999
Reintubation (*n*, %)	2 (4.3)	1 (2.1)	>0.999
In-hospital mortality (*n*, %)	1 (2.1)	1 (2.1)	>0.999

n, number; RM, retraction suspension; SM, suspension group.

### Survival

**Figure 4:**
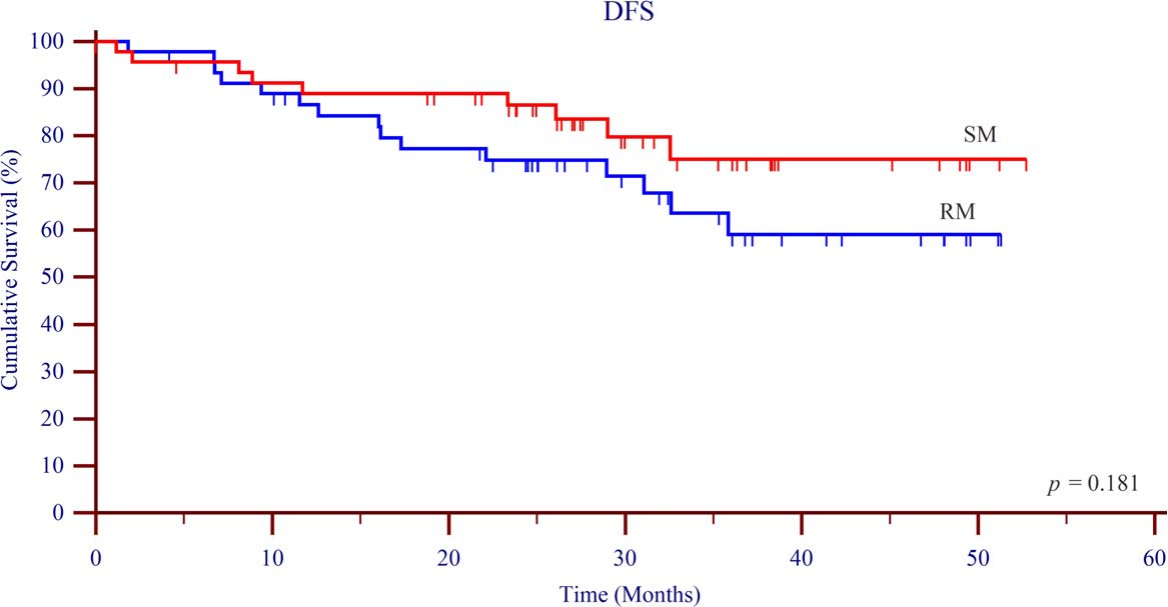
Disease-free survival. DFS, disease-free survival; RM, retraction method; SM, suspension method.

**Figure 5:**
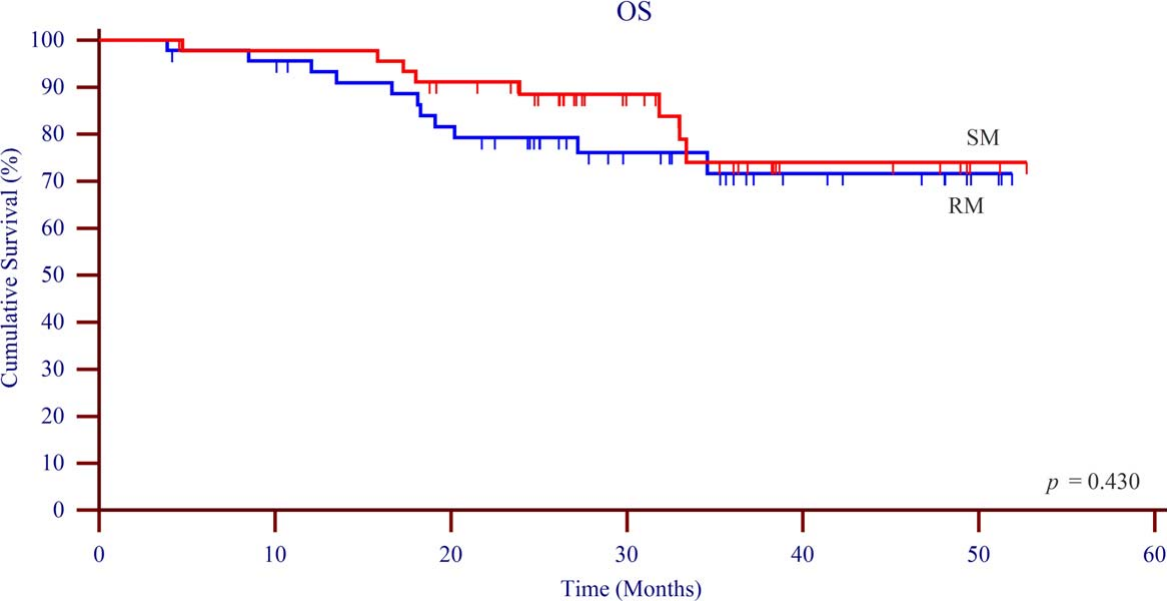
Overall survival. OS, overall survival; RM, retraction method; SM, suspension method.

Survival data of each group were compared to analyze the effect of different methods in lymphadenectomy along the LRLN on patient prognosis. The 3-year DFS (Fig. 4) rate was 80.8% in the SM group and 69.0% in the RM group (*P*=0.180). Similarly, no difference was observed in 3-year OS (Fig. 5) between the two groups (SM: 82.6%, RM: 76.1%, *P*=0.430). These observations collectively implied that the diverse methodologies in lymphadenectomy along the LRLN exerted no appreciable influence on patient prognosis.

### Quality of life

Finally, with regard to the evaluation of postoperative QoL, all surviving patients duly completed questionnaires during their postoperative outpatient clinic visits at 3, 6, and 12 months subsequent to surgery. The assessment of QoL was executed through the employment of EQ-4D-5L questionnaires. In the first 3 months postoperatively, most of the patients complained of dysphagia and reflux in both groups. With elapse of time, the symptoms were alleviated and the assessment score by EQ-4D-5L was increased. When comparing the postoperative QoL between groups, no significant difference was exhibited at different time points (all, *P*>0.05) (Table 4).

**Table 4:** Assessment of postoperative quality of life.

Different time points	SM	RM	*P*
3 months	58.128±9.997	58.6383±9.966	0.679
6 months	68.170±13.072	66.468±12.413	0.287
12 months	78.979±21.670	74.319±27.52	0.663

n, number; RM, retraction suspension; SM, suspension group.

## Discussion

The main goal of this perspective trial was to attempt to explore an optimal LRLN dissection method during the MIE for ESCC patients. While none of the results (surgical outcome, postoperative complications, survival rate, QoL, etc.) were significant differences between groups. The results of our study indicated that both methods were safe and feasible. Nonetheless, the RM emerged as more favorable than the SM in terms of curtailing surgical duration when executing lymph node dissection along the LRLN. Lymph nodes along the bilateral RLN are thought to be highly involved and complete dissection of these nodes is imperative in ESCC patients with curative intention. However, conducting lymphadenectomy along the LRLN via minimally invasive means presents formidable challenges, demanding considerable technical prowess due to the anatomical intricacies, which in turn contribute to the operative limitations. Generally, no area of thoracic surgery is more challenging or more daunting than that of lymphadenectomy along the LRLN. Some surgical outcome indicators concern the direct quality of lymphadenectomy, that is, the number of lymph node retrieval, iatrogenic injury of nerve and others are used because they are expected to be associated with long-term outcomes^9^. Moreover, this procedure is associated with considerable postoperative morbidity and impairment in healthy-related QoL with symptoms of hoarseness, dysphagia, and expectoration problems. In light of escalating standards for care quality, enhancing dissection precision while concurrently reducing postoperative morbidity and mortality assume paramount importance. Notably, the attainment of a sufficient surgical field forms the foundational prerequisite for a secure and viable lymphadenectomy along the LRLN. Differing strategies for field exposure have been documented, bifurcating into two principal approaches: esophageal division prior to lymph node dissection and lymphadenectomy without esophageal division. In order to maximize visualization of the surgical field, some surgeons described several methods by transection of the esophagus before lymphadenectomy. Akagawa *et al*.^10^ introduced a concept of mesotracheoesophagus, which facilitated to understand the surgical layer from the embryological foregut development. After mobilizing the ventral side of the esophagus from the membranous portion of the trachea and the left main bronchus, the esophagus was divided. A ʻplate-likeʼ structure including LRLN and its branches, surrounding tissues and lymph nodes, and esophageal feeding arteries can be identified with proper countertraction. Oshikiri *et al*.^5^ devised a method (ʻbascule methodʼ) for lymphadenectomy along the LRLN. In their study, the esophagus, which is considered a hindrance during lymphadenectomy, was transected at the level of the aortic arch to obtain a better surgical field. Additionally, lymph nodes dissection performed by the ʻbascule methodʼ exhibited a shorter operative duration and less estimated blood loss with an equivalent lymph node harvested compared with a conventional method. Makino *et al*.^6^ described another method (stripping method) in which the esophagus was divided together with the nasogastric tube, then the proximal residual esophagus was stripped by pulling the nasogastric tube in the revised direction and retracted toward the neck. The author announced that in stripping method, the operator can obtain a clear surgical field, enabling the safe and straightforward lymphadenectomy along the LRLN.

As mentioned above, the advantage of a divided esophagus before lymphadenectomy could increase the amount of retraction possible, allowing for further drawing and development of the surgical field. However, this process also increases the risk of the dissemination of cancer cells, especially when the tumor was located in the upper or middle third of the esophagus. Therefore, other surgeons preferred to perform lymphadenectomy without esophagus transection. It was described that after mobilization of the esophagus from the anterior and ventral side, a traction tape was used to suspend the esophagus. The lateral pedicle of the upper mediastinum including LRLN and its branches, lymph nodes, tracheoesophageal arteries, and connective tissues was extended. Meanwhile, the trachea was rotated to the right and ventral side by the assistant to help the exposure of the surgical field. After accomplishing these steps, the LRLN was easy to be recognized^11–13^. In alignment with these methodologies, our approach eschews esophageal transection in favor of lymphadenectomy through either esophageal retraction or suspension, effectively achieving equivalent surgical field enlargement. Additionally, financial considerations may deter the preference for esophageal transection.

Among the primary objectives of lymph node dissection along the LRLN is the mitigation of paralysis, a prevalent postoperative complication that carries potential life-threatening implications. The rate of recurrent laryngeal paralysis noted in our study stands at 13.8%, aligning with previous investigations (ranging from 8.3 to 40.9%)^14–16^. Lacking countertraction will result in exfoliating the lymph nodes difficult and tracheoesophageal artery bleeding, which will increase the risk of LRLN injury. Therefore, in the RM group, one of the disadvantages is that the surgeon’s left hand may be used to grasp the lymph nodes along the LRLN during the dissection. Another disadvantage in the RM group is that the assistant may touch and stretch the LRLN during the dissection in order to help the operator to explore the dissectible layer, which may result in a high-risk of LRLN injury too. This may be the reason that patients in the RM group (19.1%) have a little higher incidence of LRLN paralysis than that in the SM group (8.5%), although no statistical significance (*P*=0.231). Compared to the RM group, the lower rate of RLN paralysis may contribute to a better exposure of surgical plane, despite a longer time spent on this process. When considering other major postoperative complications, no difference was exhibited between groups either. As known, pneumonia is one of the most common complications after esophagectomy, even in minimally invasive procedures. Previous studies indicated that RLN paralysis is a high-risk factor associated with pneumonia because of aspiration^17^. Within our study, nearly half of the patients from each group who experienced RLN paralysis subsequently developed pneumonia, with approximately half of these cases necessitating reintubation. Given the profound implications of pneumonia on oncological outcomes^18,19^, the prevention of RLN paralysis assumes paramount importance.

Although the relationship between postoperative complications and survival may not be strictly causal, certain prior studies have indicated that postoperative complications could potentially disrupt the immune system and contribute to early recurrence^20–22^. To the best of our knowledge, survival status is firstly assessed in comparison to different lymphadenectomy methods along the LRLN in MIE. In the current study, a favorable result regarding survival rate was obtained when compared to the previous study, with the 3-year DFS and OS being 66.8 and 73.1%, respectively. Besides, it was found that lymphadenectomy along LRLN performed by RM has an effect on survival equivalent to SM. A hypothesis can be offered to interpret this improvement. Survival benefit can be obtained by an extended lymphadenectomy, especially when the bilateral RLN lymph node is involved. In our study, neither surgical outcome, except dissection duration, nor postoperative complication is different in each group. It is indicated that surgical skills improved according to a learning curve.

Additionally, as far as we know, QoL assessment was carried out between different lymphadenectomy methods along the LRLN for the first time. As known, esophagectomy is associated with an impairment in QoL with symptoms of reflux, dysphagia, fatigue, and vocal cord paralysis (VCP)^23–25^. After surgery, most patients experience enduring dietary changes and related symptoms for an extended period, which has a negative impact on QoL^23^. Wherefore cause this result, one of the most common reasons is the LRLN injury during the lymphadenectomy, which is responsible for temporary and permanent VCP, and has a long-lasting adverse effect on QoL. In the present study, patients in both groups had a deterioration in QoL within the first 3 months postoperatively; however, QoL will gain over time in most of the patients, especially in whom recovered from VCP. Although the result of QoL assessment between groups in the current study is robust or remained too heterogenous to further interpret, the result indicated that both methods in lymph node dissection along the LRLN are safe and feasible. Whereas the different dissection methods along the LRLN are not decisive in resulting in the pool QoL, both procedures should be optimized to facilitate continuous quality improvement in the future.

The present study has limitations. Firstly, the study was carried out in a single tertiary hospital, and all surgeries were performed by a highly experienced surgical team, which might limit the generalization of our results to others. Secondly, the study was designed from the aspect of gross anatomical viewpoints, whether the different methods can influence the meticulous anatomical dissection is still unknown. Finally, only two different lymphadenectomy methods were applied whether other methods are superior to these two remains unidentified. As such, there exists a compelling imperative for further investigation encompassing a broader spectrum of institutions and encompassing a more comprehensive array of dissection methodologies to ascertain the optimal approach to lymphadenectomy along the LRLN during MIE for patients afflicted with ESCC.

## Conclusion

This feasibility study demonstrated that both the RM and the SM in lymphadenectomy along the LRLN showed favorable outcomes and a similar safety profile to be proven in larger multicenter trials. It is noteworthy that the RM demonstrates a distinctive advantage in terms of curtailing surgical duration during the execution of lymph node dissection along the LRLN.

## Ethics approval and consent to participate

This study was conducted in conformance with Good Clinical Practice guidelines and the Declaration of Helsinki and approved by the ethics committee of the Army Medical Center of PLA (Daping hospital) (number: 201816). The patients/participants provided their written informed consent to participate in this study.

## Consent for publication

Written informed consent was obtained from the patient for publication of this case report and accompanying images. A copy of the written consent is available for review by the Editor-in-Chief of this journal on request.

## Sources of funding

None.

## Author contribution

W.G. and Y.-j.W.: conceived and designed the experiments; X.-D.H., X.-F.X., and K.-K.L.: collected the follow-up data; Y.-Q.H. and T.B.: analyzed the data; Y.-J.W., and Y.-Q.H.: wrote the manuscript.

## Conflicts of interests disclosure

The authors declare that they have no conflict of interest.

## Research registration unique identifying number (UIN)

1. Name of the registry: Chinese clinical trial registration.
2. Unique identifying number or registration ID: ChiCTR1800016963.
3. Hyperlink to your specific registration (must be publicly accessible and will be checked): http://www.chictr.org.cn/edit.aspx?pid=28828&htm=4.

## Guarantor

Wei Guo, Department of Thoracic Surgery, Army Medical Center of PLA (Daping Hospital).

## Availability of data and materials

The datasets used and/or analyzed during the current study are available from the corresponding author upon reasonable request.

## Provenance and peer review

Not commissioned, externally peer-reviewed.
